# Genome evolution in yeast reveals connections between rare mutations in human cancers

**DOI:** 10.15698/mic2014.06.153

**Published:** 2014-06-02

**Authors:** Xinchen Teng, J. M. Hardwick

**Affiliations:** 1 College of Pharmaceutical Sciences, Soochow University, Suzhou, Jiangsu Province 215123, PRChina.; 2 W. Harry Feinstone Department of Molecular Microbiology and Immunology, Johns Hopkins University Bloomberg School of Public Health, Baltimore, Maryland 21205 USA.

**Keywords:** yeast knockouts, genome evolution, secondary mutations, cancer progression

## Abstract

Cancer cells are riddled with mutations. Less than one percent of these are thought to be mutations that drive cancer phenotypes. However, a recent study conducted on the yeast knockout collections by Teng *et al.* [Mol. Cell (2013) 52: 485-494] provides hard evidence that single gene deletions/mutations in most non-essential genes can drive the selection for cancer-like mutations.

Mutations are the raw materials for evolution and disease development such as cancer progression. The vast majority of genetic mutations appear to occur by random chance due to imperfect DNA replication mechanisms, providing a mechanism for beneficial plasticity or disaster. The full extent of human genetic plasticity is unknown. Basal mutation rates are challenging to estimate, and the effects of known environmental chemical mutagens and radiation exposures on mutation rates contribute to the complexities. Even a generally agreed upon rate of around 10^-8^ changes per base per human generation (perhaps 200 changes per individual based on the genome sequences of family members) does not capture a considerable number of copy number changes of varying lengths, and largely assumes homogeneity within an individual. Mutation rates for different genome regions within a single cell also can vary greatly, and cannot be easily extrapolated from a single fertilized egg to a multi-trillion cell individual where many billions of cells die and get replaced on a daily basis. Growing trends in the field suggest the prevalence of mutations is even higher overall. Conservatively, it appears there are no two cells in the human body with the exact same sequence.

Problems arise when one of these rare mutations provides some growth and/or survival advantage that also escapes the checks and balances designed to eliminate potentially dangerous cells. Essentially all of these mutations reduce overall fitness, yet cells carrying such a mutation can become more abundant in a given niche, and logic follows that greater genetic diversity provides a greater chance that at least one cell in a population will prevail under a given selection pressure, such as chemotherapy. Best illustrated by experiments on tractable model organisms, cells with compensatory (suppressor) mutations are selected under environmental pressures such as extreme temperatures, nutrient depletion, and drug selection. However, transiently advantageous mutations can also be selected by intrinsic selection pressures such as preexisting mutations. One example is the mutator phenotype resulting from mutations in genes that preserve chromosome integrity such as DNA mismatch repair genes in cancer cells. However, it was unknown whether mutations in only a small subset of genes can drive the selection for further genetic changes without deliberate selection pressures, or if a mutation in any single gene in a given genome is capable of driving the selection for new mutations.

To address this question, we used the *Saccharomyces cerevisiae* knockout collection as a tool to ask if deletion of any non-essential gene is of significant consequence. Presuming that cell survival and cell growth rates are among the strongest drivers of evolution, we analyzed over 1000 different parental knockout strains from the haploid yeast knockout collections. For each parental strain, several single cell-derived substrains were characterized for preexisting cell death and/or growth phenotypes. We found that unlike several wild type strains, the majority of knockout strains contain phenotypically and genetically distinct individuals all sharing the same knockout gene (Figure 1A). These variant phenotypes are definitely not subtle, but may have gone unnoticed for so long because they are not detected under normal conditions (rich or minimal complete media), but are only revealed by relatively mild cell stress conditions (20 min heat ramp) or reduced nutrients (30% lower amino acids). This heterogeneity is not due to epigenetic phenomena, stochastic fluctuations in gene expression, the mechanics of knockout construction and drug selection, nor to experimental mix-ups, and all queried strains had the intended knockout gene engineered correctly.

**Figure 1:**
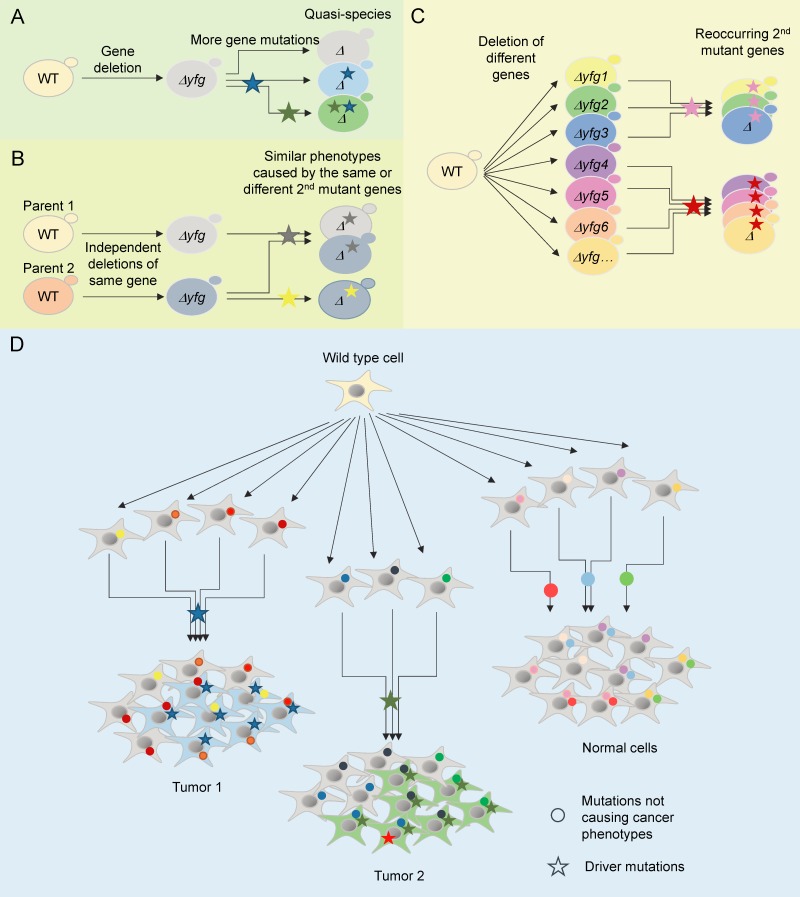
FIGURE 1: Model of genome evolution driven by gene mutation. **(A)** Most yeast knockout strains, including those lacking your favorite gene (yfg), are quasi-species harboring prevalent additional mutations, only one or two of which cause prominent cancer-like phenotypes. **(B)** Independently constructed knockouts of the same gene tend to evolve similar phenotypes by acquiring secondary mutations in the same or different genes, indicating a selection process driven by the first mutation - the original knockout. **(C)** Deletion of different genes can drive the selection for mutations in the same genes. **(D)** A potential cancer progression model. Wild type human cells acquire random mutations when replicating/repairing DNA for each cell division, most of which have no known consequence and are referred to as passenger mutations. However, occasional random mutations that have functional consequences build selective pressures for further (partially compensating) genetic alterations, some of which are cancer driver mutations and cause tumorigenesis. Hard evidence from yeast implies that mutations in a large portion of human genes have the capacity to drive the selection for cancer mutations.

Strikingly, the phenotypic variants within individual KO strains were not caused by multiple cooperating secondary mutations. Instead, all the gene knockout strains tested had only a single second mutation responsible for a given phenotype. In the few knockouts with two secondary mutations, each mutation caused a different phenotype. By applying just two assays, testing for cell death/survival following stress and for overgrowth on low nutrients, mathematical estimates indicate that deletion of any one of approximately 75% of the non-essential genes in the yeast genome has resulted in acquisition of a meaningful secondary mutation strongly affecting at least 22% of the cell population. These estimates are based on only two types of assays, sampling six substrains of each parental KO strain. Perhaps additional types of assays could reveal meaningful mutations in the remaining knockout strains.

What drove the selection for these secondary mutations? Propagation and distribution of the original yeast knockout/knockin collections to hundreds of laboratories made passaging these collections a necessity. Thus, it is expected that random mutations will have accumulated as these strains age. Indeed, knockout strains can have dozens of nucleotide changes of unknown consequence. However, if the specific secondary mutations that we found were driven in part by loss of the original knockout, then this evolutionary process should repeat in independently constructed knockouts of the same genes. We thus checked 40 pairs of knockout strains in which the same gene was independently deleted in each pair and found that 26 of these 40 pairs of knockout strains evolved the same variant phenotypes (Figure 1B). More striking, for 15 of these 26 pairs, both members of each pair evolved mutations in the same gene (functional complex) based on genetic complementation tests. Such examples of parallel evolution are extremely unlikely to occur by chance, and they strongly suggest that the original knockout played a critical role in driving the selection of secondary mutations. This evidence supports the hypothesis that a loss-of-function mutation in nearly any single gene is sufficient to build selective pressure for acquiring compensatory mutations that partially rescue the loss of the original gene. Perhaps this should not be surprising considering that genomes have evolved over millennia to their current composition.

In cancer, only about 150 genes, when mutated, are currently thought to explain the majority of human cancer phenotypes. Perhaps this situation is similar for yeast, where secondary mutations in only a subset of yeast genes are capable of compensating for the gamut of primary gene mutations. Therefore, we addressed this question by performing genetic complementation tests and genome sequencing. We found several groups of knockout strains with shared secondary mutant genes (complementation groups) (Figure 1C). Interestingly, knockout strains lacking different components of the same protein complexes, such as those involved in regulating TOR, evolved new mutations in the same gene, providing further evidence for the potency of gene-driven parallel evolution. Environmental conditions undoubtedly contributed to the evolution, but are definitely not the only important players.

What can we learn here about tumorigenesis? These studies in yeast provide hard evidence for what was suspected to occur in advanced tumors undergoing clonal expansions (Figure 1D). Unlike cancer, the first gene mutation that drove yeast evolution is known - the engineered knockout gene. The phenotypes exhibited by evolved yeast have some semblance of cancer-like phenotypes (e.g., niche-specific growth advantages with decreased overall fitness). What’s more, typically it is the secondary mutation, not the original knockout that causes these cancer-like phenotypes. We probed the available cancer genome database and found many examples of pairs of mutant genes that are homologous to the co-occurring mutant yeast genes in the same tumor. For example, mutations in a large family of human KCTD genes (yeast Whi2) often co-occurred with a mutation in STED2 (yeast SET2), one of the ~150 human cancer genes. Although several members of the KCTD gene family have been associated with various human cancers, the importance of this mutation duo in cancer is unexplored. However, given the mutation frequency of these genes in cancer, their co-occurrence is highly significant. Perhaps yeast will help overcome the difficulty in distinguishing relevant mutations from the remaining passenger mutations. The parallel evolution observed in yeast knockouts suggests that it may be possible to predict which rare mutations are potential mini-drivers that can select for the more troublesome cancer-driver mutations.

Our findings imply that a functional mutation in any single gene is enough to build selective pressures for further genetic alterations. This phenomenon reflects the interdependency of genes within a modern genome and suggests evolution can be driven quickly through several mutations. The presence of widespread secondary mutations in the yeast knockout collections reinforces the need to confirm gene-phenotype relationships. Furthermore, the new connections established by identifying pairs of co-occurring mutant genes in yeast and in human cancers will provide novel insights into biology as well as cancer progression. Identification of early genetic events before driver mutations arise in cancer could potentially help guide clinical treatment strategies.

